# Editorial: Innovations in the mental health applications of interRAI assessments

**DOI:** 10.3389/fpsyt.2022.1074794

**Published:** 2022-11-11

**Authors:** Jason Ferris, Tom Strating, Gary Cheung, Jyrki Heikkilä, John P. Hirdes

**Affiliations:** ^1^Centre for Health Services Research, Faculty of Medicine, The University of Queensland, Brisbane, QLD, Australia; ^2^Department of Psychological Medicine, Faculty of Medical and Health Sciences, School of Medicine, The University of Auckland, Auckland, New Zealand; ^3^Department of Psychiatry, Turku University Hospital, Turku, Finland; ^4^School of Public Health Sciences, University of Waterloo, Waterloo, ON, Canada

**Keywords:** person and family centered care, life course, quality of life, diseases (MeSH), psychometric, continuum of care, performance measurement quality of care

## What is interRAI?

Mental health issues concern individuals and populations in all stages of life and pose unique challenges to healthcare systems. People with mental health conditions are exposed to complex interactions between psychological, biological, social, and environmental influences that are unlikely to be mitigated by one-dimensional assessment and screening systems. The interRAI research collaborative—www.interrai.org—aims to improve the quality of life of people of all ages, particularly those who are vulnerable due to some combination of age-related or developmental health problems, disability, medical complexity, or mental health challenges. The collaborative does this by designing and implementing comprehensive systems that cross the continuum of health and social care settings. Since Morris et al. (1) first described the deployment of a single-sector *Resident Assessment Instrument* (RAI) for geriatrics in response to the US Omnibus Reconciliation Act of 1987, interRAI instruments have evolved to become a fully integrated suite of measures spanning populations of all ages. Researchers and health professionals use interRAI systems in more than 35 countries for care planning, outcome measurement, resource allocation, quality improvement, and policy development.

This Frontiers in Psychiatry special issue presents a compilation of research to illustrate the novel *mental health* (MH) applications of the interRAI suite in psychiatric and non-mental healthcare settings. Today, more than a billion people experience mental health disorders, accounting for 19% of years lived with disability (2). While persons living with mental health disorders or addictions have diverse needs throughout their lives, healthcare services are frequently uncoordinated; often failing to holistically meet the needs of community. InterRAI instruments provide a highly validated mental health information system with a whole-of-person approach to mental health assessment (3). Emergency departments, inpatient psychiatry units, community mental health services, mobile crisis teams, police officer screening, and long-term care settings internationally use interRAI systems for all persons across the life span (4).

## Measuring the mental health impacts of COVID-19

The sudden uncertainties of the COVID-19 pandemic intensified the existing challenges faced by strained mental healthcare systems worldwide. Betini et al. assessed the mental health impact of the pandemic on a general population survey in Canada. Using interRAI's self-reported mood scale, the authors gathered data on 3,127 individuals about their mental health in four online surveys spanning April to July 2020. The number of study participants feeling anxious and depressed increased more than two-fold compared with a pre-pandemic survey iteration. Nevertheless, the authors emphasize that these sobering statistics are dynamic and can change rapidly in response to social change.

Research from Stewart, Vasudeva, et al. indicates that neither adults nor children were spared from the life-changing consequences of the COVID-19 pandemic. Stewart, Vasudeva, et al. examined longitudinal routine care data collected from 35,000 children using interRAI's *Child and Youth Mental Health Assessment* (ChYMH). Paradoxically, the researchers found sharp declines in the number of children and youth referred to mental health services during lockdowns. This disparity exposes a need to increase children's access to mental healthcare in times of crisis.

In a subsequent paper, Stewart, Celebre, Semovski, et al. highlight the versatility and applicability of interRAI's ChYMH in a fragmented children's mental healthcare system unable to supply the demand for child mental health services. The review elucidates that the lack of coordination between the numerous mental health professionals involved drives inefficiency. The authors state that by taking an integrated approach to assessing a child's strengths, needs, and preferences, interRAI's suite of child and mental health assessment instruments provides an evidence-informed solution to these problems across sectors (Stewart, Celebre, Semovski, et al.).

A paper by Hirdes et al. focused on the development of new measures of mood disturbance with a sample of about half a million individuals. The contexts ranged from the general community-based population to persons in long-term care and palliative care programs. The paper demonstrates the feasibility of large-scale consistent measurement of mood across populations with differing levels of health, functional ability, and cognition. A remarkable result was that the level of severely distressed mood during the pandemic was seven times greater in the general population compared with a pre-pandemic sample. This level of distress approached what was seen in clinical populations receiving community mental health services.

Mental health problems in the workplace are prevalent worldwide, but the needs of injured workers who receive psychiatric services remain elusive (5). A Canadian observational study by Herring et al. used the richness of data collected between 2006 and 2016 from the interRAI MH instruments to provide a unique insight into the needs of this distinct population. Concerningly, the authors found that workers experienced more trauma, pain, depression, sleeping issues, and substance use disorders than other psychiatric inpatients. Herring et al. emphasize the importance of ongoing interRAI measurements to capture and respond to the symptoms and needs of a growing patient population.

## Mental health and quality of life

A major focus of interRAI assessment addresses quality of life (QoL). Persons with mental health disorders typically have a lower QoL. Hence, a deeper understanding of this multidimensional concept could generate new avenues for targeted interventions to improve the lives of vulnerable persons. Celebre et al. investigated determinants of QoL in children and youth receiving mental health services. Using a combination of the ChYMH and the innovative QoL-ChYMH, the authors found that specific mental state indicators had a disproportionate influence on QoL scores. For instance, participants who experienced anhedonia and depressive symptoms scored significantly lower on the social domain of QoL. In addition, individuals experiencing heightened depressive symptoms also reported having lower QoL at the individual (e.g., autonomy) and basic needs (e.g., food) levels.

Research by Luo et al. and de Almeida Mello et al. examined QoL of adults in mental health settings with a seven-nation study including low, mid, and high resource countries using the *Self-Reported QoL Survey for Mental Health and Addictions* (SQoL-MHA). Given interRAI's seamless integration of items and scales across all its assessment tools—including those for inpatient and community-based mental health services—the study was able to measure QoL's objective and subjective realms. Participants from Canada and Finland scored particularly high for the hope and activities dimension. On the other hand, patients from Rwanda, Belgium, and Brazil reported good relationships with staff. The findings suggest that strength-based international collaboration could benefit patient's quality of life.

## Instruments for an aging population across the continuum of care

Older adults constitute the fasted growing age group, with the estimated number of people aged 65 years and over exceeding 727 Million (6). Older adults constitute a vulnerable population with elevated levels of mental health or substance use disorders. Home care has emerged as a viable strategy to reduce hospital or long-term care institutionalization. Poss et al. examined how many community-dwelling older adults had psychiatric diagnoses and other mental health symptoms and what proportion of these patients visit a psychiatrist. Responses to the interRAI Home Care data showed that only a quarter of participants visited a psychiatrist, despite more than half having psychiatric diagnoses. The authors highlight important questions about differential access to psychiatry services by site of care, geographical location, and age.

Once institutionalized, frail older adults become exposed to institution-acquired complications and interventions such as infection, malnutrition, and control interventions with adverse physical and psychological effects. Using routinely collected data from 200,000 interRAI MH assessments, Cheung et al. examined determinants of control interventions (e.g., physical or chemical restraint) in inpatient psychiatry. Their research highlights that people with functional impairment, psychosis, aggressive behavior, cognitive impairment, and delirium were at risk of controlled interventions in non-emergency situations. Considering that these can have negative health effects, the authors advocate for other strategies to support older adults in these situations.

On an optimistic note, research from Howard et al. shows that long-term care settings provide person-centered care to an increasingly inclusive population of disabled and medically complex persons. Their study analyzed longitudinal data from the third-generation interRAI Minimum Data Set to determine if the nursing home transition toward person-centered care continues in today's diverse patient landscape. Although less conducive to social wellbeing, the authors conclude that person-centered care in US nursing homes provides the necessary foundation to promote mental and physical wellbeing for persons with complex needs.

An emerging body of literature indicates that resilience, the positive mood response observed in response to stress or adversity, promotes wellbeing in older adults (7). In a world-first study, Angevaare et al. used routine care data collected from older Dutch residents of long-term care facilities to explore the mental health effects of different psychological stressors. The interRAI dataset enabled the authors to compare associations between both observer and self-reported mood outcomes. Remarkably, the study found that in their Dutch sample, major life stressors, particularly conflict with other care recipients and staff, were associated with positive mood symptoms.

Measuring changes in cognition over time is crucial for the early detection and treatment of cognitive impairment in older adults. The InterRAI *Cognitive Performance Scale* (CPS)—ranging from 0 (intact) to 6 (very severe impairment)—and the *Montreal Cognitive Assessment-5 min* protocol (MoCA 5-min)—ranging from 0 to 30—are frequently used for measuring cognition in long-term and clinical care settings. Since older adults frequently move between these settings, Andersson et al. were able to link scores on both instruments to facilitate the tracking of cognition across the continuum of care. The authors found that a CPS score of 0 (intact) and 3 (moderate) corresponds to a MoCA 5-min score of 24 and 0, respectively. This study demonstrates the opportunity of cross-walking scores between the two cognitive measures; however, the authors noted that CPS had higher sensitivity for severe cognitive impairment, whereas the MoCA 5-min was superior for measuring mild impairment.

## New additions to the mental health suite

While many articles in this special issue draw on existing data extracted from interRAI assessments, three publications explored the psychometric evaluations of new interRAI mental health instruments. First, Barbaree et al. describe the development of a forensic supplement to the *MH* and *Problem Behaviour Scale*. The instrument underwent rigorous evaluations in three samples of adult forensic inpatients, prison inmates, and youth in custody. The authors state that their innovative tool enables mental health professionals to predict which inpatients are at risk of violence across forensic settings. In practical application, early identification could facilitate appropriate treatments to reduce the need for acute control interventions and to manage behaviors that could hinder the person's progress toward community reintegration.

Stewart, Celebre, Hirdes, et al. designed an algorithm to predict violence specifically in children and youth. The authors deemed the interRAI Risk of Injury to Others (RIO) algorithm a strong predictor of violence that can competently assist decision-making and facilitate early intervention. Lastly, Stewart, Celebre, Iantosca, et al. designed and validated a novel *Autism Spectrum Screening Checklist* (ASSC). The authors report that the ASSC can serve as an initial screen to identify high-risk children and youth assessed as part of routine practice.

## Next steps for the interRAI mental health suite

In summary, the articles in this Research Topic provide valuable insights into the richness of interRAI data and recent mental health innovations within the interRAI suite of assessment systems. These data are not exclusive to interRAI fellows, but researchers interested in exploring further the value of the data are encouraged to contact the authors or visit our website. Moreover, these studies and the new innovations support the needs and outcomes of persons with mental illness across different care settings, age groups, and countries.

## Author contributions

JF, GC, JH and JPH contributed to the framework of the editorial and draft reviews. TS drafted the editorial. All authors contributed to the article and approved the submitted version.

## Conflict of interest

The authors declare that the research was conducted in the absence of any commercial or financial relationships that could be construed as a potential conflict of interest.

## Publisher's note

All claims expressed in this article are solely those of the authors and do not necessarily represent those of their affiliated organizations, or those of the publisher, the editors and the reviewers. Any product that may be evaluated in this article, or claim that may be made by its manufacturer, is not guaranteed or endorsed by the publisher.
